# Successful mechanical thrombectomy for terminal ICA bullet embolism: A case report

**DOI:** 10.1177/15910199231219021

**Published:** 2023-12-10

**Authors:** Jared Clouse, Bart Thaci, Austin Sy Clark, Michael Baggett, Osama Raslan, Charles Matouk, Ben Waldau, Branden Cord

**Affiliations:** 1 Department of Neurosurgery, 1439 University of California, Davis, Sacramento, CA, USA; 2 Department of Neurosurgery, 6595 University of Pittsburgh Medical Center, Harrisburg, PA, USA; 3 12218 School of Medicine, University of California, Davis, Sacramento, CA, USA; 4 Department of Radiology, 12224 University of California, San Francisco, CA, USA; 5 Department of Radiology, 21772 University of California, Davis, Sacramento, CA, USA; 6 Department of Neurosurgery, 12228 Yale University, New Haven, CT, USA

**Keywords:** Thrombectomy, bullet fragment, bullet embolism

## Abstract

Bullet embolism after high velocity penetrating trauma is a rare event that can have devastating and wide-ranging effects distant from the original site of injury. A 29-year-old presented with multiple gunshot wounds to the chest, back, abdomen, and lower extremities but no penetrating head injury. After proper resuscitation, the patient was noted to have left-sided hemiparesis and computed tomography angiography of the head showed a bullet fragment that had traveled to the right M1 segment of the middle cerebral artery resulting in occlusion of the vessel. Mechanical thrombectomy was performed in an attempt to remove the bullet fragment but this was unsuccessful as the fragment was firmly lodged in the blood vessel. Aspiration of clot distal to the fragment was then performed in hopes of preventing a large volume ischemic event which was angiographically successful resulting in TICI 2c revascularization. This case demonstrates that thrombectomy can be safely and successfully performed distal to a lodged foreign body.

## Introduction

Bullet embolism is a rare event resulting from penetrating trauma when a bullet, pellet, shrapnel, or projectile penetrates a single wall of a blood vessel without exiting the opposing vessel wall and is subsequently carried to a location distant from site of initial injury. These events rarely involve the intracranial vasculature as there are very few blood vessels that are large enough to transit an embolus of that size. They are limited to injuries to the pulmonary veins, carotid arteries or would be possible in patients with a patent foramen ovale. In cases involving the cerebral vasculature, many different treatment methods have been employed including, open surgery, endovascular procedures, and conservative management, with varying results.^1^ This is also only the third occurrence in the literature where endovascular removal of an intracranial bullet embolus has been attempted, with only one successful removal of the bullet fragment.^1,2^ Herein we describe the first documented case of endovascular mechanical thrombectomy distal to an embolized bullet fragment, without removal of the foreign body.

## Case presentation

### History

A 29-year-old male patient presented to the emergency department with 14 gunshot wounds to the chest (2), abdomen (7), back (1), and bilateral lower extremities (4). Upon arrival the patient was hemodynamically unstable with labored breathing but was awake, following commands and moving all extremities. A chest tube was placed in a large pneumothorax, and the patient was taken to the operating room for exploratory laparotomy prior to any neurosurgical evaluation. Intra-operatively, the patient had multiple injuries to his liver and bowel, but no thoracic exploration was performed as the patient stabilized with chest tube placement and repair of viscera injuries. Post-operatively, the patient remained intubated but followed commands with grossly full strength on the right and no movement on the left when the sedation was held. Due to the decreased movement on the left side of his body, a computed tomography angiogram (CTA) of his head and neck was urgently obtained.

### Imaging

**Figure 1.**
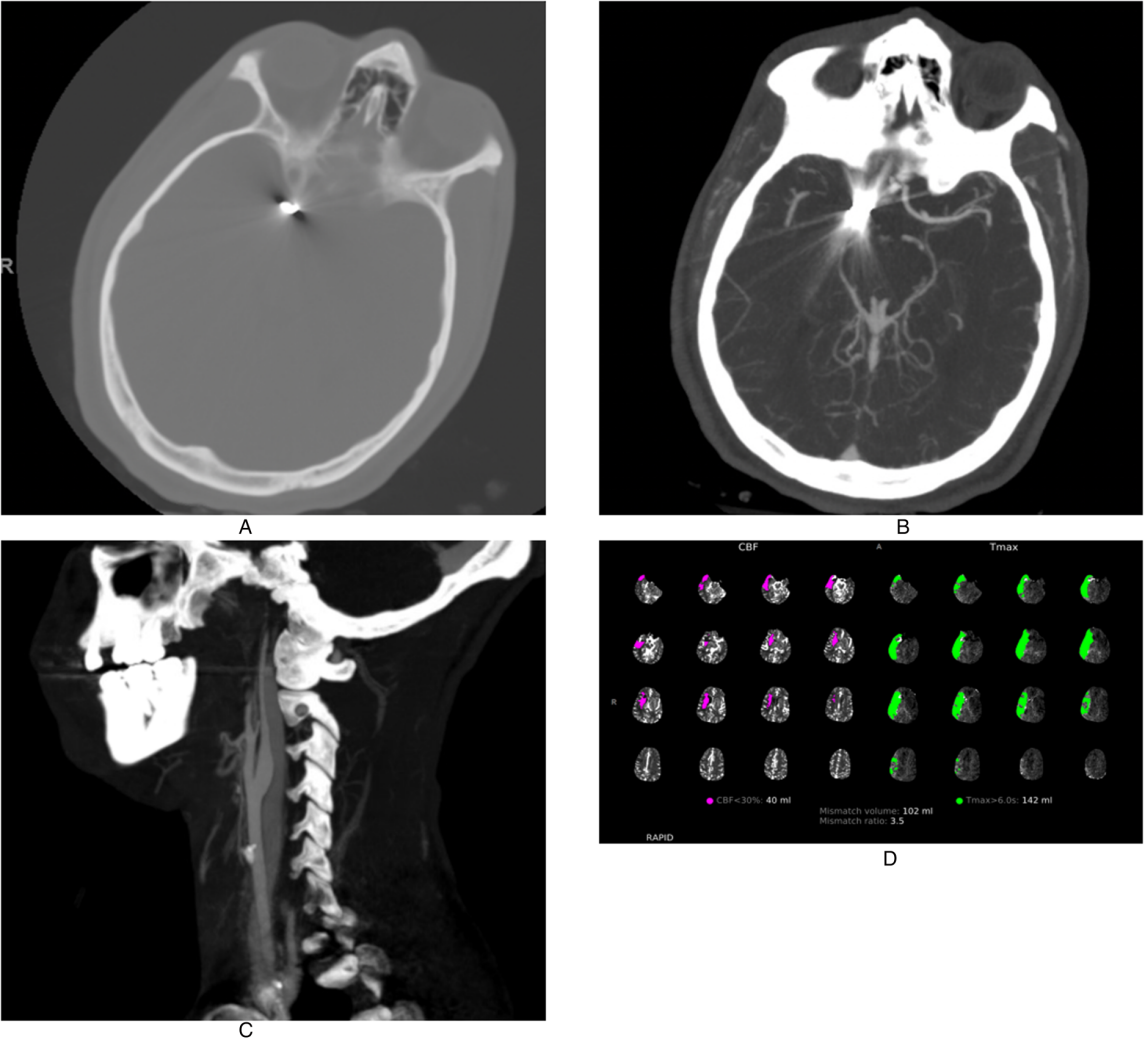
(A) CT head demonstrating metallic fragment with intact calvarium. (B) CTA head showing filling defect in right MCA. (C) CTA neck demonstrating ICA vasospasm starting at the level of C3. (D) Perfusion scan showing large mismatch between penumbra and core infarct.

**Figure 2.**
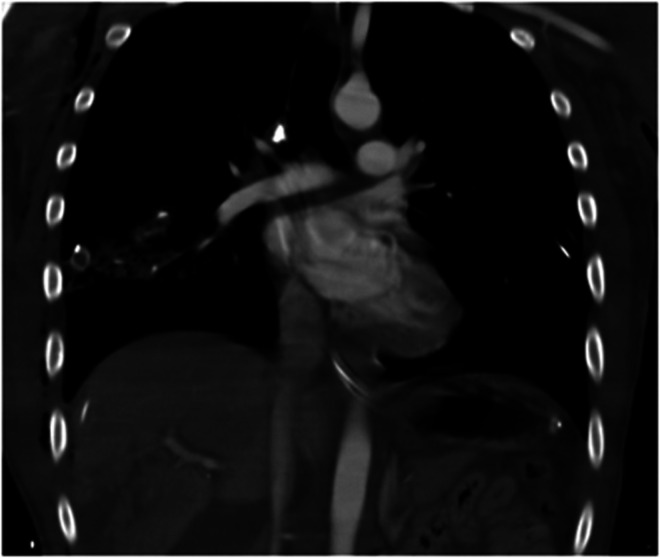
CT chest showing trauma to the right lung with a chest tube in place. Additionally there is a tract from the bullet in communication with the pulmonary vasculature.

CTA of the head demonstrated a metallic foreign body lodged at the right internal carotid artery (ICA) terminus with an intact calvarium and no evidence of intracranial hemorrhage or trauma (Figure 1(A)). Vessel imaging showed occlusion of the M1 segment of the middle cerebral artery (MCA) with distal reconstitution of the MCA vasculature, although this was obscured by streak artifact from the metal fragment (Figure 1(B)). Additionally a smooth stenosis of the right ICA from the level of C3 to the petrous portion of the ICA was also appreciated concerning for possible vasospasm secondary to the travel of embolic metal fragment (Figure 1(C)). CT perfusion of the brain was subsequently obtained and demonstrated a large 102 ml mismatch between penumbra and core infarct (Figure 1(D)). Due to the high volume of salvageable brain, endovascular retrieval of the presumed bullet fragment and mechanical thrombectomy of the MCA thrombus were attempted. Review of initial imaging showed bullet fragments adjacent to the pulmonary vasculature on the right and this was the presumed site of embolization of the bullet fragment (Figure 2).

### Endovascular intervention

**Figure 3.**
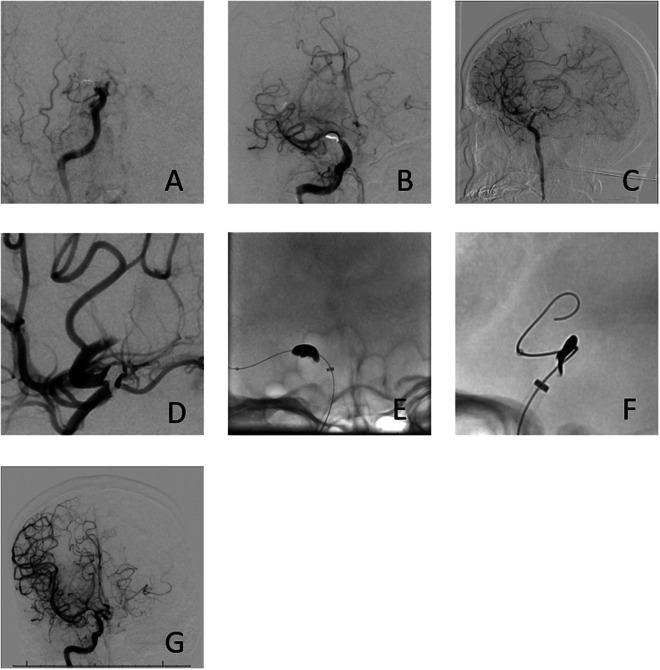
(A) Right common carotid artery injection with no filling of ACA or MCA. (B,C) Right selective ICA injection showing bullet fragment, M2 occlusion, and periocallosal artery occlusion. (D) Magnified view of bullet fragment showing two channels created in the ICA by the fragment. (E,F) Navigating microcatheter and aspiration catheter distal to bullet fragment. (G) TICI 2c recanalization with persistent pericallosal artery occlusion.

The patient was taken to the interventional radiology suite where a right femoral sheath was placed and 90 cm BMX96 guide catheter was positioned in the right common carotid artery, where initial injection showed ICA filling to the level of the supraclinoid ICA with no filling of the MCA or ACA (Figure 3(A)). Selective catheterization using a velocity microcatheter over a Syncho2 soft 014 microwire of right ICA was performed and subsequent injection showed filling of the bilateral ACAs as well as filling of the MCA on the right. However, filling defects were noted in the right pericallosal artery as well as the inferior division of M2 (Figure 3(B) and (C)). Additional injections showed that the fragment was thin and flat causing bifurcation of the parent vessel into two smaller channels (Figure 3(D)). A Sofia Flow Plus aspiration catheter was then gently brought to the face of the metallic fragment and multiple attempts were made to remove the fragment with aspiration. These attempts proved to be unsuccessful and the fragment was felt to be firmly lodged in place and additional attempts could potentially disrupt the parent vessel and aspiration of the fragment was aborted. Additionally, there was also concern that dislodging the fragment could cause tearing of the vessel wall and irreparable damage due to the sharp edges of the fragment. For the same reason, no stentriever mechanical thrombectomy or snare removal were attempted. A large open channel was noted adjacent to the bullet fragment and attention was then turned to the occluded inferior division of M2. An Aristotle 018 microwire and a Penumbra 3MAX aspiration catheter were advanced distal to the fragment a large clot was aspirated without restitution of distal flow (Figure 3(E)). A second aspiration was performed where additional clot burden was removed. Post-thrombectomy angiogram showed TICI 2c revascularization of the right MCA territory (Figure 3(F)). There was however persistent occlusion of the right pericallosal artery. Due to the orientation of the bullet fragment, the right A1 segment was not able to be catheterized and the attempt was abandoned.

### Outcome

Post-operatively the patient's neurologic exam remained stable with a dense left-sided hemiplegia. The patient was started on a continuous heparin infusion immediately post-thrombectomy to prevent further clot formation secondary to the retained bullet fragment. There was bolus given when the heparin drip was started and the drip was titrated to an Anti-Xa goal of 0.3–0.5. On the first day post-operatively, the patient stopped following commands and his right pupil was noted to be dilated and non-reactive. Urgent head CT showed contrast staining in the right basal ganglia and increased edema with increased midline shift (8 mm). The heparin infusion was stopped and reversed using protamine and the patient was taken for emergent hemicraniectomy. After surgery the patient was following commands on the right with no movement on the left. Head CT obtained two weeks after presentation showed completion of right MCA and ACA territory strokes, likely due to reinfarction after the heparin drip was stopped to facilitate the hemicraniectomy, with resolution of midline shift due to brain herniation through the hemicraniectomy site. The patient remained in the ICU for three weeks post-operatively but was eventually extubated and transferred to the inpatient rehabilitation service. The patient returned to the OR four months after his initial procedures for replacement of his bone flap. At that time the patient had regained some motor function in his left lower extremity (3/5 strength with hip flexion and knee extension and 1/5 strength in ankle plantarflexion and dorsiflexion) but was still unable to ambulate independently and he had no return of function in his left upper extremity.

## Discussion

Intravascular embolization of metallic fragments is a rare complication after penetrating injury. While there have been over 250 cases of documented missile embolization in the literature, only 52 of these cases have involved the cerebral vasculature.^3,4^ Shotgun pellets are by far the most common object to embolize to the brain, occurring in 40 of the 51 documented cases.^1^ It has been theorized that shotgun pellets are able to embolize more easily than metallic fragments due to their small round shape and as they enter the patient at a relatively lower rate of speed compared to other metallic projectiles. These characteristics could potentially allow the pellet to enter but not exit a blood vessel and roll through the vessel more easily than a fragment with an irregular shape which is more likely to become lodged more proximally.

Due to the rarity of embolized bullet fragments there is no consensus on how to treat this condition. The majority of cases (32 of 52) have been treated conservatively without neurosurgical intervention.^5^ Many different surgical techniques have been employed including craniotomy with and without open arteriotomy with fragment extraction, ECA to ICA bypass, and endovascular aspiration of the fragment with no particular treatment modality showing superior clinical outcomes. Only on two previous occasions has endovascular aspiration of a shotgun pellet been attempted with successful aspiration occurring in one case, but aspiration of a bullet fragment has never been attempted.^1,2^

Endovascular approaches to embolized bullet fragments were not attempted prior to 2018 but now as technology has advanced the use of endovascular techniques, it could become a preferred approach for these rare occurrences. This case represents the third attempted endovascular aspiration of a metallic fragment embolized to the cerebral circulation which was ultimately unsuccessful. However, this case was able to demonstrate the feasibility and safety of a mechanical thrombectomy distal to a lodged foreign body.

Performing a thrombectomy distal to a lodged foreign body is high risk and likely only plausible in a few select cases. There are many risks in navigating a catheter distal to a sharp foreign object including risk of the catheter getting sheared or lodged, the clot getting sheared off during removal and damage to the parent vessel. The use of different types of aspiration catheters and possibly balloon guide catheters could be helpful but would need to be assessed on an individual case basis. In this case there was a sufficient corridor through a relatively large channel adjacent to the bullet fragment which allowed for small aspiration catheter to be advanced to the face of the clot and perform the thrombectomy.

The thrombectomy was a success from a technical standpoint with a TICI 2c revascularization. It is unknown if the postoperative heparin infusion would have been sufficient to prevent clot formation throughout the patient's hospitalization or long term. Unfortunately, the patient developed cerebral edema requiring surgery and precluding the use of anticoagulant therapy which eventually lead to completion of a right ICA stroke.

## Conclusion

Embolized metallic fragments to the intracerebral circulation after penetrating injury is incredibly rare. There is no current consensus for how to treat this extremely rare phenomenon but herein we present an endovascular approach for attempted fragment aspiration and mechanical thrombectomy distal to the fragment. As endovascular techniques continue to improve removal of these foreign bodies endovascularly, they become more practical and faster than an open approach. Although our patient progressed to a complete ICA stroke, we demonstrated the feasibility of mechanical thrombectomy in the setting of a lodged foreign body.

## References

[1] Nussbaum ES Graupman P Goddard JK , et al. Air gun orbitocranial penetrating injury: emergency endovascular treatment and surgical bypass following pellet migration to middle cerebral artery: case report. J Neurosurg Pediatr 2018; 21: 270–277. 29271732 10.3171/2017.8.PEDS17320

[2] Hassan AE Rabah RR Tekle W . Intracranial pellet embolization: an endovascular endeavor. BMJ Case Rep 2019; 12. DOI:10.1136/bcr-2019-015301 PMC6954796 31892630

[3] Kuo AH Gregorat AE Restrepo CS , et al. Systematic review of civilian intravascular ballistic embolism reports during the last 30 years. J Vasc Surg 2019; 70: 298–306.e6. 30922763 10.1016/j.jvs.2019.02.004

[4] Bahnini A Petitjean C Kieffer E . Gunshot pellet embolus to the middle cerebral artery. Ann Vasc Surg 1986; 1: 139–142. 3333005 10.1016/S0890-5096(06)60716-4

[5] Wang H Ning XJ Chen C , et al. Surgical treatment of foreign body embolus in the middle cerebral artery secondary to neck injury. Br J Neurosurg 2020; 34: 512–517. 30696273 10.1080/02688697.2018.1556781

